# Adverse reactions in venom immunotherapy protocols: conventional versus ultra-rush

**DOI:** 10.1080/07853890.2022.2112969

**Published:** 2022-09-16

**Authors:** Ali Selcuk, Abdullah Baysan, Sait Yesillik, Fevzi Demirel, Ozgur Kartal, Mustafa Gulec, Ugur Musabak, Osman Sener

**Affiliations:** Department of Immunology and Allergy, Gulhane Training and Research Hospital, Ankara, Turkey

**Keywords:** Hymenoptera venom allergy, safety, insect sting, effectiveness

## Abstract

**Background:**

Venom immunotherapy (VIT) is an effective treatment in the patients at high risk of anaphylaxis or life-threatening systemic reactions due to Hymenoptera venom allergy. But, systemic and large local reactions can be observed, especially during the build-up phase of VIT. We evaluated the safety of conventional and ultra-rush build-up protocols.

**Materials and methods:**

Two protocols in 71 patients (39 conventional and 32 ultra-rush protocols) with honeybee and wasp venom allergy were evaluated retrospectively. Patients were diagnosed and selected for VIT according to the criteria established by the European Academy of Allergy and Clinical Immunology. The severity of systemic reactions was evaluated according to the criteria of Mueller.

**Results:**

Build-up phases were tolerated in 66.2% (*n* = 47) without any reaction. Allergic adverse reactions were observed in 33.8% (*n* = 24): large local reactions 22.5% (*n* = 16) and systemic reactions 11.3% (*n* = 8). There was no significant difference in the number of adverse reactions comparing patients receiving conventional and ultra-rush protocol. In addition, no association was found between allergic adverse reactions and the following factors: sex, previous systemic sting reactions, honeybee and wasp venom extract.

**Conclusion:**

We found that both protocols were tolerated in patients with honeybee and wasp venom allergy. Ultra-rush protocol will be preferred for patients and clinicians because of its advantages in terms of time and costs.

**KEY MESSAGES**
- VIT is the only curative treatment method that reduces the risk of severe reactions after a bee sting and improves the quality of life in patients with Hymenoptera venom allergy.
- Ultra-rush VIT protocol has advantages such as few injection and time savings.
- Both ultra-rush and conventional VIT are safe treatments to prevent potentially life-threatening reactions in patients with honeybee and wasp venom allergy.

## Introduction

Hymenoptera venom allergy (HVA) is an immunoglobulin E (IgE)-mediated disease and may present with clinical manifestations ranging from mild systemic reactions such as generalized cutaneous symptoms to severe systemic reactions such as cardiac or respiratory arrest [1]. In epidemiological studies, it has been reported that systemic reactions are seen in 0.3–7.5% of adults [2]. In addition, HVA is the most common cause of anaphylaxis in adults in Europe [3].

Venom immunotherapy (VIT) is the only safe and effective curative treatment approach that reduces the risk of systemic reactions in patients with HVA. It is known that, in the general population, honeybee venom immunotherapy is effective in 77–84% of the patients, and wasp venom immunotherapy is effective in 91–96% [1,4]. VIT is administered by subcutaneous injections and consists of build-up and maintenance phases to ensure a sustained effect. There are different protocols for VIT applications [1], including conventional and ultra-rush protocols that we often apply in our clinic. Compared to conventional protocol, ultra-rush protocol has obvious advantages such as shorter build-up time, fewer hospital visits, optimum patient compliance, and less labour and time waste [1,5–7]. However, there are some concerns with the ultra-rush protocol regarding adverse reactions [1,8].

Although VIT is generally well tolerated by patients, adverse reactions such as large local reactions (LLR) and life-threatening systemic reactions (SR) can sometimes occur. These adverse reactions are usually IgE-mediated and are observed especially during the build-up phase [1, 8, 9]. Although there are studies in the literature reporting that the ultra-rush protocol is safe [5–7], the EAACI guidelines and the US Practice Parameters indicate that the risk of systemic reaction is higher in rush/ultra-rush protocols [1,10].

This study aims to compare the frequency and severity of adverse reactions during the build-up phases of conventional and ultra-rush VIT protocols.

## Materials and methods

In this retrospective study, treatment cards and visits records of patients aged ≥18 years who received VIT in the Allergy and Clinical Immunology Unit of Gulhane Training and Research Hospital were evaluated through patient files. Patients receiving angiotensin-converting enzyme inhibitors and beta-blockers were switched to an alternative drug in all patients before VIT. This study was accomplished according to the guidelines of the Helsinki Declaration and verified by the Clinical Research Ethics Committee of Gulhane Training and Research Hospital, Ankara, Turkey (approval number: 01.07.2014/40).

### Diagnostic procedures

The previous systemic sting reaction evaluations of the patients were performed according to H.L. Mueller [11]. In addition, HVA diagnoses and eligibility assessments for immunotherapy were made according to the criteria determined by the European Academy of Allergy and Clinical Immunology (EAACI) [1,12]. Prick and intradermal skin tests with honeybee and wasp venoms were performed (ALK-Abello, Hørsholm, Denmark or Alyostal, Stallergenes, Antony Cedex, France), specific IgE for both venoms were measured (positive cut-off ≥0.35 kIU/L, ImmunoCAP system Thermo Fisher, Uppsala, Sweden). Serum tryptase levels were measured in 6 of 8 patients who developed SRs during the build-up phase of VIT (ImmunoCAP Tryptase, Thermo Fisher, reference range <11.4 µg/L).

### VIT protocols

Conventional and ultra-rush VIT protocols (at 30-min intervals) were performed. Ultra-rush build-up phase of immunotherapy was performed with a venom dose of 0.1, 1, 10, 20, 30, 40 µg (cumulative total dose of 101.1 µg). The patient was seen again after 1 week (day 8) and given 50 µg in each of two injections and subsequently returned after 1 week (day 22) for an injection of 100 µg. Conventional protocol consisted of 17 injections (one weekly) with cumulative dose of 332.14 µg (0.02 µg, 0.04 µg, 0.08 µg, 0.2 µg, 0.4 µg, 0.6 µg, 0.8 µg, 2 µg, 4 µg, 6 µg, 8 µg, 10 µg, 20 µg, 40 µg, 60 µg, 80 µg and 100 µg). In all VIT protocols, patients' intravenous lines were placed, and vital signs were recorded. Routine antihistamine and/or corticosteroid administration for pre-treatment was not performed. The purified aqueous preparations Alyostal (Stallergenes, Antony Cedex, France) were administered during the ultra-rush protocol. Conventional protocol was performed with depot extracts Alutard SQ (ALK-Abelló, Hørsholm, Denmark).

### Adverse reactions

All injections were administered subcutaneously to the mid-lateral or posterior region of the upper arm. Erythema and swelling ≥10 cm at the injection site were considered as large local reaction. Systemic reactions were evaluated according to H.L. Mueller [11]. After the adverse reactions were treated, the same protocol was continued from the step that the patient could tolerate.

### Statistical analysis

Data were analyzed with the SPSS© v20.0 software (IBM, Chicago, IL. Licence number 10240642). Distribution of the numerical variables were tested by the Kolmogorov–Smirnov test. Mann–Whitney *U* test, Chi-square test, and Fisher’s Exact test were used to make univariate comparisons. Data were presented as mean ± standard deviation (SD), min.–max. values, frequencies, and percentages using 95% confidence limits. *p*-values <.05 were considered as statistically significant.

## Results

Data of 71 participants were analyzed. Of the participants, 39 (54.9%) had received conventional VIT, and 32 (45.1%) ultra-rush VIT. Most participants (66.2%, *n* = 47) had no adverse reactions, while 22.5% (*n* = 16) had LLR and 11.3% (*n* = 8) had SR.

There were no significant differences in the mean age or sex of the conventional and ultra-rush groups. Also, there were no differences between the groups regarding the Mueller grade or venom type (Table 1).

**Table 1.** Comparison of the conventional and ultra-rush immunotherapy build-up groups concerning demographics, Mueller grade and venom type.

	Conventional (*n* = 39)	Ultra-rush (*n* = 32)	Total (*n* = 71)	Test	*p*
Age (mean ± sd)	35.2 ± 10.6	37.7 ± 13.9	36.3 ± 12.2	0.659*	.510
Sex, *n* (%)					
Female	11 (28.2)	6 (18.8)	17 (23.9)	0.863^#^	.353
Male	28 (71.8)	26 (81.2)	54 (76.1)		
Mueller grade, *n* (%)					
I	4 (10.3)	2 (6.2)	6 (8.5)	2.078^$^	.587
II	13 (33.3)	7 (21.9)	20 (28.2)		
III	15 (38.5)	14 (43.8)	29 (40.8)		
IV	7 (17.9)	9 (28.1)	16 (22.5)		
Venom type, *n* (%)					
Honeybee	22 (56.4)	13 (40.6)	35 (49.3)	1.752^#^	.186
Wasp	17 (43.6)	19 (50.4)	36 (50.7)		

*Mann–Whitney *U* test, ^#^Chi-square test, ^$^Fisher–Freeman–Halton test.

In our ultra-rush protocol, all adverse reactions (SR and LLR) were observed on the first day. The presence of adverse reactions and adverse reaction subgroups LLR or SR were similarly distributed between the conventional and ultra-rush groups (Table 2).

**Table 2.** Comparison of the conventional and ultra-rush immunotherapy build-up groups regarding adverse reactions, large local reaction (LLR), and systemic reaction (SR).

	Conventional (*n* = 39)	Ultra-rush* (*n* = 32)	Test	*p*
Adverse reactions, *n*(%)			1.212^#^	.271
No	28 (71.8)	19 (59.4)		
Yes	11 (28.2)	13 (40.6)		
LLR, *n* (%)				
No	33 (84.6)	22 (68.8)	2.534^#^	.111
Yes	6 (15.4)	10 (31.2)		
SR, *n* (%)				
No	34 (87.2)	29 (90.6)	0.209^$^	.722
Yes	5 (12.8)	3 (9.4)		

*All adverse reactions on the first day, ^#^Chi-square test, ^$^Fisher’s exact test.

No significant differences were observed between the different groups from the perspective of adverse reactions (Table 3). Furthermore, the mean ages of the participants with and without adverse reactions were 36.2 ± 12.1 (*n* = 24) and 36.4 ± 12.3 (*n* = 47). There were no significant differences in the mean ages of participants with and without adverse reactions (independent-samples *t*-test = 0.070, *p* = .945). The clinical characteristics of the patients who developed SRs during the build-up phase of VIT are presented in Table 4.

**Table 3.** Comparison of the groups regarding adverse reactions.

	Adverse reactions					
	No		Yes			
	*n*	%	*n*	%	Test	*p*
Sex						
Female	11	64.7	6	35.3	0.022^#^	.882
Male	36	66.7	18	33.3		
Mueller grade						
I	5	83.3	1	16.7	1.534^$^	.677
II	14	70.0	6	30.0		
III	17	58.6	12	41.4		
IV	11	68.8	5	31.2		
Protocol						
Conventional	28	71.8	11	28.2	1.212^#^	.271
Ultra-rush	19	59.4	13	40.6		
Venom type						
Honeybee	21	60.0	14	40.0	1.185^#^	.276
	26	72.2	10	27.8		

^#^Chi-square test, ^$^Fisher–Freeman–Halton test.

**Table 4.** Clinical characteristics of patients with systemic reactions during the build-up phase of VIT.

Patient	Age, y	Sex	Protocol	Venom type	Sting reaction, Mueller grade	Extract dose of SR, µg	SR, Mueller grade	Serum tryptase (µg/L)
1	45	Male	UR	HB	3	20	1	None
2	51	Male	UR	HB	3	40	2	None
3*	40	Male	UR	HB	4	10	3	4.5
4	19	Male	C	Wasp	2	20	2	4.8
5	32	Male	C	HB	2	40	2	5.7
6	39	Male	C	HB	3	40	2	8.4
7*	56	Female	C	Wasp	3	20	3	4.3
8*	24	Male	C	HB	4	80	3	6.6

HB: honeybee; UR: ultra-rush; C: conventional; SR: systemic reaction; *patients administered adrenaline.

## Discussion

In multicenter studies, the frequency of systemic reactions with VIT ranges from 8% to 20% [1,8,13,14]. 11.3% of our patients developed SR. There was no significant difference in SR rates between the ultra-rush protocol and the conventional protocol (9.4% versus 12.8%, respectively; *p* = .722).

In our ultra-rush protocol, all adverse reactions (SR and LLR) were observed on the first day we reached a cumulative dose of 101.1 µg. Birnbaum et al. found the SR rate 11.1% during the build-up phase of the ultra-rush protocol, and the mostly of SRs were observed on the first day (cumulative dose of 101.1 µg) [5]. Roll et al. studied a total of 67 patients who underwent ultra-rush immunotherapy and reported the SR rate 12.5% (all on the first day, cumulative dose of 111.1 µg) during the increase in dose [15]. Rueff et al. compared build-up phases of ultra-rush and conventional protocol and reported the SR rates 11.4% and 4.3%, respectively. But, the rates reported in this study included severe systemic reactions (according to Ring and Meßmer grade III-IV) [8]. On the other hand, Korosec et al., found the SR rate 38.7% during the build-up phase of ultra-rush protocol but their patients received treatment only with honeybee venom [16]. In general, treatment with honeybee venom involves risk for SR [5,7,8,17–19]. However, honeybee was not found as a risk factor in some studies [6,15]. In our study, the rate of SR was higher in patients who received honeybee venom immunotherapy than wasp venom immunotherapy, but the difference was not statistically significant (17.1% versus 5.6%, respectively; *p* = .151).

The vast majority of systemic reactions due to VIT are mild or moderate [5,11–19]. In our study, 3 patients (1 ultra-rush, 2 conventional) who developed SR required a single dose of adrenaline. In the other 5 patients, antihistamine ± corticosteroid treatment was sufficient. We measured serum tryptase levels in 6 of 8 patients and continued the same VIT protocol.

In our study, LLR occurred in 22.5% of patients. In addition, the rate of LLR was higher during the ultra-rush protocol than the conventional protocol (31.2% versus 15.4%, respectively; *p* = .111). In our study, ultra-rush was performed with a purified aqueous preparation, while conventional was performed with a depot extract. In general, purified aqueous preparations tend to cause more LLR than depot extracts [4,20].

In the study conducted by Roll et al., the LLR rate was 5% during the build-up phase of the ultra-rush protocol [15]. Similarly, Cosme et al., found the LLR rate 5.4% during the build-up phase of the ultra-rush protocol [19]. All patients in these studies received pre-treatment with antihistamines two or three days before and on the morning of ultra-rush itself [15,19]. In our study, the patients did not receive pre-treatment, therefore LLR rates may be higher than previous studies. Also, we did not find a significant relationship between LLR rates and venom types (honeybee group: 22.9%, wasp group: 22.2%; *p* = .949). All LLRs were treated with topical corticosteroids and oral antihistamine.

The main limitations of our study were that it was retrospective and was conducted with a small group of patients. However, the majority of the studies in the literature investigating the adverse reactions of VIT seem to have similar limitations. Prospective studies with large patient groups are needed to determine possible risk factors for these adverse reactions that develop during VIT. Another limitation of our study was that basal serum tryptase levels were not measured at the time of beginning VIT. Therefore, it was not possible to reveal a possible relationship between adverse reactions and basal serum tryptase level.

## Conclusion

We found that rates of adverse reactions were similar between ultra-rush and conventional protocol. In addition, there was no difference in frequency and severity of adverse reactions between honeybee and wasp venom. The majority of adverse reactions were mild and responded easily to properly treatment. In the light of these data, ultra-rush immunotherapy protocol, with its many advantages, can serve as first-line treatment for honeybee or wasp immunotherapy.

## Data Availability

The data based on the results of the current study were obtained, are accessible from the corresponding authors upon reasonable request.
